# Zoom and its Discontents: Group Decision Making in Pediatric Cardiology in the Time of COVID (and Beyond): Aurthors

**DOI:** 10.1007/s10916-023-01944-1

**Published:** 2023-05-05

**Authors:** Mark H.D. Danton, Ian Bushnell

**Affiliations:** 1 Department of Paediatric Cardiac Services, Royal Hospital for Children, 1345 Govan Road, G51 4TF Glasgow, Scotland, UK; 2 School of Psychology and Neuroscience, University of Glasgow, University Avenue, G12 8QQ Glasgow, Scotland, UK

**Keywords:** COVID-19, Group decision-making, Video-conferencing, Virtual meetings, Congenital heart disease

## Abstract

The emergence of Covid-19 has led to change within hospital-based healthcare. An example, has been to reconfigure clinical decision making meetings from traditional in-person (Face-to-face, FtF) to online video-conferencing (VC) format inorder to decrease contagion risk. Despite its widespread uptake, there is minimal empirical data evaluating this format. This narrative review considers the implications on medical decision-making when clinicians communicate remotely via Microsoft Teams. The discussion is informed by the psychological literature and by commentary obtained from a survey of paediatric cardiac clinicians who participated in clinical meetings when video-conferencing was first introduced. Whist video-conferencing can optimize clinician presence, this is potentially offset by compromises in current imaging quality, the group discussion, information sharing and decision quality. Implementing a shift from face-to-face to VC within the group decision-making process requires an appreciation of the changed environment, appropriate adaptations and the implemention of new technology solutions. Meanwhile, healthcare should carefully consider the potential implications of clinical decision making using online video conferencing, be prepared to adapt and evaluate prior to a shift away from face-to-face formats.

Hospital based healthcare has had to adjust to the constraints imposed by the COVID-19 pandemic. Nonclinical staff, including management and administration, employed working-from-home with online communication to decrease the risk of viral spread. By contrast clinical teams continued to provide ‘hands-on’ patient care safeguarded by various infection control measures such as PPE (personal protective equipment), vaccination, self-testing, and contact tracing with home isolation. Remote working has been implemented in the clinical environment to manage certain activities including outpatient services [1]. But arguably one of the most significant changes to clinical working has been in the reformatting of clinical meetings, where decisions on patient management shifted from face-to-face (FtF) to online. Whilst the pandemic has resolved such practice continues.

Conversations with paediatric cardiac clinical staff led to the development of a localised staff survey to evaluate possible concerns about the impact of virtual working. This survey surfaced opinions that deserve further consideration. This article therefore considers practical implications for group medical decision-making, in the context of the multidisciplinary meeting, when undertaken by clinicians who are communicating remotely through online platforms such as Zoom and Microsoft Teams. The discussion is informed by the psychological literature on group decision-making and illustrated by commentary obtained from paediatric cardiac clinicians from our department who participated in meetings during the pandemic when remote videoconferencing (VC) was first adopted. Because of the range and variability of diagnostic categories contained within congenital heart disease it is one of the most complex and challenging areas of medical practice [2]. Thus, clinical decisions tend to be inherently multifaceted and can involve significant patient risk. The spectrum of clinical decision making can be broadly divided into two types: judgments involving ratings or estimations, and decisions involving choices amongst discrete alternatives.

Today, overarching decisions on specialist patient care are taken through multidisciplinary team meetings (MDTs) and traditionally these meetings are held face-to-face [3]. These meetings comprise a group of clinicians with varied and relevant expertise, who collectively review clinical information, discuss and formulate decisions on individual patient care. The MDT utilises ‘*the wisdom of the crowds’* paradigm recognising that decisions made by a group will in general be superior to that made by an individual expert within the group [4–7]. The aggregation of varying judgments increases accuracy as individuals’ errors tend to cancel out at group level. Decision-making by individuals is also prone to heuristics, bias, fallibility, and irrationality [8–10], and by capturing a wider viewpoint these cognitive flaws can be mitigated. Further, the diverse thinking of a group offers the advantages of differing perspectives, the emergence of new ideas, and co-construction of new knowledge through collaboration and co-operation [11].

Although virtual MDTs have been used prior to Covid-19, largely to accommodate scheduling and the geographic dispersion of clinicians [12], the pandemic has acted as a catalyst to increase their widespread use [13]. In the main, previously established clinical teams who have used traditional FtF MDTs prior to the pandemic, transitioned to virtual formats. With some teams this virtual condition has been employed exclusively where all participants are communicating online. But in the majority a hybrid MDT model, combining FtF with videoconferencing, has been the preferred format. The hybrid MDT operates as a spoke and hub model. The hub represents the physical meeting room located within the hospital which is fully equipped for case presentation and display of imaging data. Resident in this room are the chairperson and a varying number of clinicians who host the meeting while adhering to COVID guidelines of social distancing and mask wearing. At the spokes reside the online clinicians who remotely view presentations and participate in discussion through the online VC platforms of MS Teams or Zoom. The transition from FtF to the virtual MDT has brought new opportunities and introduced challenges, specifically with viewing imaging data, group discussion and decision aggregation. Despite being widely adopted, there is uncertainty, and limited empirical data, on whether virtual MDT can maintain the quality of decision-making provided by FtF [14].

Judgements along a continuum are often required to inform clinical decisions. For example, quantifying the strength of heart contraction or the degree of cardiac valve dysfunction is based upon clinicians viewing and grading moving images from echocardiographic and cardiac magnetic resonance scans. With FtF meetings, these scans are presented to the group using high quality projection with an accompanying narrative provided by an imaging specialist. All clinicians view the same images. At the request of group members, key images can be re-presented if diagnostic uncertainty arises. Remote clinicians visualise these images via internet connections using computer or phone screens that vary in quality. Insufficient bandwidth and image resolution can lead to sub-optimal viewing of a rapidly contracting heart. Under these conditions the online clinician may not see the images adequately, and opt out from the assessment. In this situation, the diagnostic conclusions defaults to the hub located clinicians who are interpreting the higher quality imaging at source. By reducing the number of participants contributing to the assessment the accuracy and reproducibility are likely to be compromised. This has been previously demonstrated with radiological detection of breast cancer and with interpretation of ECGs, where diagnostic accuracy improves with increasing group size, a phenomenon referred to as collective intelligence [15, 16]. Furthermore, online clinicians may be unwittingly persuaded by the host’s interpretation of the images, particularly if they perceive their own image quality as inferior. Misjudgements by the host may go uncorrected or become potentially exacerbated as remote clinicians pay credence to the host’s analyses. This situation is akin to Solomon Asch’s line-judgement experiments in which participants yielded to the incorrect judgement of a counterfeit majority on around 40% of occasions [17]. Despite privately querying the judgement, many accepted the error often citing doubt in their own visual interpretation. In the hybrid MDT scenario, the host’s minority position may become dominant and influential. In our department, quality of diagnostic imaging when viewed remotely was a principal concern. The imaging was described as ‘*poor and often non-diagnostic’* or ‘*substantially poorer* [compared to FtF] *on MS Teams’*. The majority of clinicians considered viewing diagnostic imaging to be worse with remote compared to FtF meetings.

Following presentation of the clinical and imaging data, group discussion occurs. Here, there is the opportunity to interrogate and clarify specific aspects of the clinical data at a deeper, more granular level. Different treatment options are proposed and pros and cons of each alternative discussed. This process is dynamic and interactive and terminates when the group achieves an implicit consensus, around a preferred treatment option: further investigations, continued medical care or surgery, etc. The aggregation of individual decisions follows a majority/plurality model which has been shown to perform well in natural group settings [18]. Recent psychosocial theories and research propose that the quality of group decision is determined by how the group processes information and their motivational characteristics [19, 20]. Within this framework, well-established errors of group decision making, including hidden profiles, social loafing and groupthink [21], can be explained by these overarching determinants of information processing and motivation. Group decision making has adapted over the eons of human evolution and in the main functions well. However, if the environmental condition surrounding the decision-making process should change significantly, then these group procedures can become maladaptive, and result in poor decisions with potentially serious adverse consequences [22]. In this regard, online participation would qualify as a fundamental change in the MDT environment which may impact on the quality of clinical decisions.

The information required for optimal clinical decisions is diverse and includes knowledge of the patient’s wellbeing, their preferences, and the clinicians’ expertise and experience of the various treatment options under consideration. Some of this information will be fully shared within the group, while other relevant facts may be uniquely held by one group member, for example the patient’s current wellbeing. If this knowledge remains unshared then this is likely to negatively impact on the quality of the group’s decision, a situation referred to as the hidden profile effect. The group’s ability to bring to the fore and share all relevant information influences the likelihood of reaching an optimal decision [23, 24].The hidden profile effect has been demonstrated in FtF clinical teams, where incorrect diagnoses were significantly more likely when discussing cases containing hidden profiles compared with control cases [25]. Because virtual teams process information differently from FtF, sharing knowledge and decision quality might be expected to be altered, a priori. Interestingly, Lu in a meta-analysis of the hidden profile paradigm identified that compared to FtF, computer mediated teams were equivalent in sharing unique information and in decision making quality [23]. However, Curşeu et al., in their systematic review on how virtual teams process information, identified that although virtual teams can be better at sharing information they have problems using and integrating information due to reduced group cohesion [26]. It is noteworthy that compared with the FtF the ability of online clinicians to contribute to the discussion in our departmental survey was deemed to be compromised. A significant proportion rated online to be inferior to FtF when evaluating meeting participation and group discussion citing ‘*difficulty in following continuity of* conversation’, ‘*disengagement in the process’* and *‘lack of active participation’.*

Social loafing describes the tendency for individuals to contribute less than their full potential when working in a group [27]. Consequently, the performance of the group may be adversely impacted compared to when all members are fully engaged and contributing. Kaba et al., proposed that under certain situational contexts social loafing can undermine the collaborative process involved in medical decision-making leading to poor outcomes [28]. Virtual collaboration has been reported to exacerbate social loafing behaviour [29–31]. Blaskovich [32] reported that compared with FtF, virtual collaboration negatively impacted on both individual contribution and overall group performance, and that social loafing may partially explain this result [32]. In their study, virtual participants displayed lower levels of participation, reduced effort and lower information recall compared to FtF participants. Group performance, as measured by the quality of decision-making, was also significantly worse with virtual compared to FtF participation [32]. The perception of social loafing can also occur in virtual teams where team members may suspect or assume that their peers are loafing because they cannot directly see their behaviour [33].Monzani et al., reported that the negative impact of perceived social loafing, diminished group cohesion and dissatisfaction with the work processes and results, was more pronounced with virtual teams compared with FtF teams [30].

With the hybrid MDT model, remote participants using Zoom or MS Teams can choose to have their cameras off and this creates uncertainty as to the degree of participation of these non-visible, on-line clinicians. For example, our clinicians expressed that they were ‘*unable to know the level of engagement of those attending virtually especially as cameras are universally off’ or* whether *‘he/she is present [in the meeting*]’. Diminishing of the fidelity of the communication media (participants with their cameras off) is likely to exacerbate social loafing, or its perception. It also eliminates important cues from facial expression and body language with potentially unfavourable consequences to group communication and their clinical decisions.

Groupthink, where highly cohesive groups exhibit premature consensus seeking that leads to poor decision making, arises when group members avoid dissent or speaking out against decisions in order to maintain or promote group harmony [34]. The hallmarks are lack of critical thinking, concurrence seeking and excessive loyalty to the group. There is an increasing interest and awareness that medical teams and MDTs may be particularly vulnerable to groupthink [6, 21, 22, 28]. This is because of the homogeneity of membership, insularity of the profession, and the embedded hierarchical behaviours within medical teams. Controversially, Kaba et al., has questioned the emphasis on teamworking in medical decision-making, arguing that this too may compromise the process [28]. The principal determinant of groupthink is strong group cohesiveness particularly in the setting of high stress pressurised environments [35]. It has been proposed that compared with collocated FtF meetings, virtual teams should be less prone to groupthink due to the reduced social contact engendering less group cohesion. However, traditional peer groupthink seems not to be the main issue within virtual teams, but instead these teams are susceptible to hierarchical groupthink where the meeting leader can exert an excessive and disproportionate influence [36]. The leader can become so dominant that the information disseminated to the team is filtered through the lens of the leader’s perceptions creating a disproportionate influence on the final decision. [27, 37].

MDT decision-making shares similarities to jury judgements [38] in so far as there are typically no ‘correct’ solutions (i.e., non-intellective tasks). However, clinical teams differ fundamentally from jury members in that they are composed of highly specialist and interdependent members, whereas jury members are homogenous and interchangeable [39]. MDT participating clinicians are tasked with estimating the prognosis of the individual patient, to consider the risk-benefit of various treatment options and then select a preference. The task is complex due to the heterogeneity of patients and their conditions, the variation in medical opinion between specialities and the changing, and often limited, evidence base. For example, Kee et al., reported large variation among the clinicians participating in lung cancer MDT with regard to estimating patient 6-month survival, treatment choice and even the fundamental aim of treatment (prolonging life, maximising the quality of life) [40]. However, this study also identified that following group discussion, modest improvements in prognostic accuracy at the group level were observed [40]. The dynamics of the group and how well the clinicians interact and communicate might be expected to influence the group performance and quality of their decisions. Therefore, the ability to see group members collocated in the same room, allowing the transfer of nonverbal and other social cues, should enhance the collaborative processes. Virtual teams using videoconferencing may closely replicate the FtF experience but the interaction tends to be less dynamic, interactive and less efficient compared to FtF [41].Rajasekaran et al., reported that in a survey of clinicians contributing to a musculoskeletal oncology MDT, 30% rated ‘depth of discussion’ and ‘interaction with specialists’ inferior when using VC compared to FtF [42], similar to the findings from our own survey.

However, social interaction can introduce biases to the team influencing their decision-making; individual judgements, behaviour and attitudes can change as a result of the peer pressure and presence of others [39]. Group interaction may polarise people, amplify their individual biases and lead them to support a course of action despite the evidence that it might be failing [39]. It is possible, as with groupthink, that social distancing afforded by online participation may reduce these biases allowing clinicians to promote and express independent cognitions, and not be swayed by the opinions or judgements of their peers.

An major advantage of online VC over FtF is in the transcending of space and time limits, the opening up to a wider audience and diversifying the membership. This is particularly relevant for highly specialist centralised services, such as paediatric cardiology, where regional clinicians outwith the institution may contribute to the decision-making. Furthermore, online VC enables communication between institutions for second opinions and referral for supra-regional specialist care e.g. cardiac transplantion. The scheduling flexibility of online meetings is advantegous in conveneing unscheduled MDTs for urgent/emergency patients where there is a time imperative for a decision.

Healthcare systems continue to evolve to meet the needs of modern practice. New applications for the storage and widespead access of clinical imaging data using cloud-based technologies are becoming available, which are data secure and NHS compliant [43]. Such technologies can support virtual MDTs by providing distributed access of high-quality imaging data to all clinicians irrespective of their format of participation. Furthermore, these can be adapted to meet specialist need of advanced visualisation required for congenital heart disease.

## In conclusion

Group-decision making through multidisciplinary teams is an essential component of modern healthcare. In providing remote access to multi-disciplinary meetings online communication has enabled clinician participation while minimising the contagion risk during the Covid-19 pandemic. Despite the many expressed shortcomings of virtual participation, the majority of our clinicians favoured the availability of a hybrid format for post-pandemic MDT meetings primarily due to the greater opportunity to access the meeting. It is important not to conflate the effectiveness of the process with its practical implementation. As a new generation of clinicians emerges, attuned to online technologies and a preference to incorporate remote working, online meetings within healthcare will likely continue and develop. However, no communication media can yet replicate the high quality of collocated human interaction. As the media richness declines, so does the quality of image perception, group interaction and potentially group decision-making. Undoubtedly near-future technologies will advance to address some of the current limitations of video conferencing and accessing high-quality imaging data. Those systems that preserve important non-verbal cues and enhance social presence should facilitate interactive behaviour and promote optimal working practices. Concurrently, as virtual work-groups gain the experience of working together they will learn to develop strategies and evolve social behaviours that adapt to this new environmental condition [44]. At present the evaluation of the virtual MDT effectiveness has been limited to survey reports [42, 45] with an absence of empirical studies comparing these alternative modes of MDT functioning. Therefore, healthcare should carefully consider the implications of online group decision making, adapt accordingly and evaluate prior to replacing the in-person face-to-face multidisciplinary meetings.

## Data Availability

Not applicable.
